# Replantation, Transplantation and Implantation

**Published:** 1905-06-15

**Authors:** J. F. Austin


### REPLANTATION, TRANSPLANTATION AND IMPLANTATION.

BY J. F. AUSTIN, D.D.S.

Read before the Southwestern Michigan Dental Society, April 12, 1905.

It is with a great deal of hesitation and reluctance, that, as a younger member of this society, I presume to give expression to my primitive ideas on this subject, and I hope you will pardon me if I do not shed any new light on this subject.

Replantation, transplantation and implantation is a subject that should receive, in my judgment, a great deal more attention than it does.

Replantation is an operation for the replacing of a tooth which has been accidentally dislodged, or, it may be, intentionally extracted for the purpose of cutting off the apex of the root for the cure of a chronic alveolar abscess, or the removing of some foreign body from the apex or end of root, such as a part of a broach or a mass of gutta-percha which has been forced through the apical foramen in filling the root canals.

A tooth when once out of its socket, even if replaced in a few minutes after its dislodgment, will never be other than a foreign body. However, if properly sterilized and the canal properly filled, it will remain in place for a considerable period and give useful service, providing that it is securely immobilized.

Some writers hold to the idea that the peridental membrane becomes regenerated, but I hardly think this is true, for until there is a partial destruction of the root there can be no fixation of the fang, as it is by the solution of a part of the lime salts of the cementum that this condition is brought about.

The process of absorption takes place at points on the circumference of the root probably through the agency of the osteoclasts, very similar to the process operating in the absorption of the roots of deciduous teeth.

If the process of absorption be rapid, then the time the tooth will remain stable is very short, but if the process is slow, its usefulness will be prolonged to a corresponding degree.

In some instances there will be no absorption at all, in such cases the tooth never becomes fixed, although it may have been thoroughly sterilized and immobilized by mechanical means. This may be owing to some condition of the root or because of mal-trea ment prior to its reinsertion. I think it always good practice to replace any tooth which has been accidentally removed, for we can count on its doing good service under favorable circumstances for a number of years. Three years ago I replaced two teeth that had been knocked out, after three or four days, and they are still in excellent condition.

I have made a great many replantations with excellent success. About three months ago a lady came to my office with trouble in a superior bi-cuspid. I examined the tooth and found it had been treated, I asked the patient if some other practitioner had been treating the tooth and she said there had for some time, but the tooth still continued to grow worse, the indications were that she had an alveolar absceess, so I proceeded to treat for that trouble. But after treating it for over three weeks, I came to the conclusion that there was something besides an abscess. I advised the patient to have the tooth extracted and I would make a replant, which she consented to have done. I extracted the tooth and found my brother practitioner in opening up the root canals, had accidentally opened through the root into the surrounding tissues and instead of broach passing up into the root canal, it had been forced through this opening into the tissues, causing a severe inflammation. I replaced the tooth as soon as possible and a few days ago I had an occasion to examine the tooth and found it apparently firm, with the surrounding tissues as healthy as any other part of the mouth.

To replant a tooth, first remove the clots of blood from socket as soon as possible after the tooth has been extracted, after which I always pack socket with some good antiseptic. I prefer oil of cloves for this purpose. This being done, scrape the root and take out the pulp, and fill the root canals. Then resect the end of the root and invest tooth in some good material for the filling. After the filling is in place, remove the tooth, clean well and render aseptic by dropping it in a small dish of oil of cloves, letting it remain there until the socket is ready for its reception. I am always very careful not to touch the tooth after this, always handling with sterilized pliers.

It may not be necessary to handle these cases with such care, but I remember a case at a clinic while I was in college, when my room-mate and myself induced a very dear friend of ours to have an elongated central incisor replanted. Our instructor made the operation, and I remember he handled the tooth with his fingers entirely. He scraped the root with an old jack knife, and did not sterilize the tooth at all. In three days the patient lost the tooth by suppuration, and we boys lost a dear friend.

I do not think we can handle these cases with too much care. After sterilization of both tooth and socket, the tooth is forced into place and secured to adjoining teeth by a fine silver or copper wire. I have the patient come about twice a week to see that the wires are still secure. I also force oil of cloves up underneath the free margin of the gum with an orange wood stick. The tooth should be sufficiently fixed in from three to four weeks to dispense with the wire.

Transplantation is rarely desirable in these days of porcelain crowns and bridges. If the root has lost its utility as a carrier of a crown it may be extracted and another tooth from another mouth be put in its place. There are several difficulties in the way of transplantation; first, the disinclination of one to have the tooth of another person in his mouth. then again the transmission of disease makes the operation more undesirable, another objection is the difficulty of getting suitable shaped teeth and properly shaped roots to fit the socket, although if the root is not of suitable shape to fit the socket, the socket or root can be modified to suit the case. However, if much of the root is cut away, the chances of its lasting or its permanency is materially decreased. The method of transplantation with the exception of changing the root or socket is the same as replantation.

Replantation and transplantation have been practiced for a long period of time, but implantation is comparatively new.

The late Dr. Wm. M. Morrison, of St. Louis, Mo., was the first to practice implantation, later Dr. Younger gave prominence to this operation. Implantation, as its name indicates, contemplates the taking of a tooth from another mouth and placing it in a socket in the process which has been made by cutting into the bone. Such teeth are put in place in the jaw when nature has failed to give the proper complement or when a tooth has been extracted and the socket has been filled by the process of nature. In other words, a new socket has been cut and a tooth from another mouth implanted.

The objection of carrying a tooth from one mouth to another holds in this case as in transplantation, the only advantage in this case over transplantations are that the sockets can be shaped to the root and that teeth implanted remain longer fixed in the jaw than either replanted or transplanted teeth, but the difference in this respect is only trifling. All go in time in exactly the same manner. Special instruments are adapted for this operations and are as follows: A bistoury, a spear-shaped drill, cross-cut fissure and round burs of various sizes, and large flamed-shaped burs. A cruciate incision is made in the gums over the site of the missing tooth, the four laps of gums are dissected up and with a spear-shaped drill, the bone is drilled to the length of the root to be inserted, care being taken that the inclination of the shafts of the drill corresponds to that of the adjoining teeth, next the socket is enlarged by the fissure burs to adapt to the root.

The socket is cleared of blood and bone debris, the tooth inserted and retained by the method before described. The opening in the gums should be a trifle less than the size of the root in order that the root may fit more closely and act as a barrier to the ingress of micro-organism and the secretions.

During the process of making the socket, the root should be occasionally inserted as a guide to the adaptation of the opening to the shape of the root.

Implantation is done only in incisors, cuspids, and perhaps, occasionally, in the bi-cuspids.

### DISCUSSION.

Dr. George W. Cook: This method of practice seems to come and go as a question for discussion in dental societies and as practice by our profession. It is a valuable procedure, and as the essayist has said, it deserves more attention from the general practitioner than it gets. Now and then some one makes a hobby of it for a time, but it could be used more frequently to advantage than it is. Why do these teeth fail after about so long? This is a question that needs answering. If we knew its answer, we could enter on its practice with more assurance of prolonging the existence of replanted teeth, and we should know when to undertake the operation and when to do something else. I have made a number of operations with the usual success. In some necrotic and abscessed conditions it is a good practice. I have also used it advantageously in pyorrhea cases.

Dr. Rowley: Once a patient came to me with a lower molar tooth which had been replanted and which had become so loose that the lady could pick it out and again replace it with her fingers. She was very anxious to have it fixed so it would not hurt her gum, as the roots had become quite rough. I amputated the roots and replaced them with hard black vulcanite. The patient was greatly pleased and wore the tooth for many years.

Dr. S. M. Fowler: I have heard of rubber necks, but this is the first time I ever heard of putting rubber roots on natural teeth. I don't like the idea of holding implanted teeth with wire ligatures; they should be securely and rigidly held in place by suitable splints, cemented to the adjoining teeth. It is a good subject to discuss and also a good one to practice occasionally.

Dr. Zederbaum: Ten years ago, when in the navy, I extracted a lower molar for a shipmate and replanted it with no antiseptic precautions to speak of. I saw it seven years afterwards and it was doing good service and as firmly planted as any other teeth.

Dr. James Doyle: A patient came to my office several years ago and I noticed a tooth that was turned one-half round in its socket. Upon inquiry, I found that ten years before the patient had gone to a dentist to have a tooth extracted and in his absence the office boy undertook the operation, with the result that he extracted this tooth by mistake and replanted it, but in doing so changed its position.

The tooth was doing good serivce. I undertook to replant a tooth in the same mouth, but it was a dismal failure in two months.

Dr. Rix: I have essayed these operations many times with both success and failure. Forty-three years ago I extracted a root on which I had placed a crown. After cleaning the root I replanted it and it remained in good service for twenty-five years. Have tried it several times with more favorable prospects and failed. Twenty-three years ago a boy had his four incisors knocked out by a pump handle. After they had been out six hours, I replanted them and they are still in place, and are of good color and the gum condition is as good as it could be. I have transplanted some teeth but never got a long service from any of them. I can't account for the difference in results.

Dr. Austin: It will be found necessary to stay the posterior teeth with metal splints, cemented to the adjacent teeth. The anterior teeth can easily be made secure by silver wire ligatures. I have somtimes used only the silk ligature. I have only practiced this work for four years, which may not be sufficiently long for me to strongly advocate this method. In that time I have never lost a tooth.

